# Fusion *DNAJB1::PRKACA* in Non-Fibrolamellar Cancer Cases. Reply to Arif et al. Global Re-Analysis Confirms Absence of the *DNAJB1::PRKACA* Fusion in Hepatoblastoma. Comment on “Fleifil et al. DNAJB1-PKAc Kinase Is Expressed in Young Patients with Pediatric Liver Cancers and Enhances Carcinogenic Pathways. *Cancers* 2025, *17*, 83”

**DOI:** 10.3390/cancers18060918

**Published:** 2026-03-12

**Authors:** Yasmeen Fleifil, Ruhi Gulati, Katherine Jennings, Alexander Miethke, Alexander Bondoc, Gregory Tiao, Rebekah Karns, Lubov Timchenko, Nikolai Timchenko

**Affiliations:** 1 Division of General and Thoracic Surgery, Cincinnati Children’s Hospital Medical Center, Cincinnati, OH 45229, USA; yasmeen.fleifil@cchmc.org (Y.F.); ruhi.gulati@cchmc.org (R.G.); alex.bondoc@cchmc.org (A.B.); greg.tiao@cchmc.org (G.T.); 2 Department of Neurology, Cincinnati Children’s Hospital Medical Center, Cincinnati, OH 45229, USA; katherine.jennings@cchmc.org (K.J.); lubov.timchenko@cchmc.org (L.T.); 3 Department of Gastroenterology, Hepatology & Nutrition, Cincinnati Children’s Hospital Medical Center, Cincinnati, OH 45229, USA; alexander.miethke@cchmc.org (A.M.); rebekah.karns@cchmc.org (R.K.); 4 Department of Surgery, University of Cincinnati College of Medicine, Cincinnati, OH 45229, USA

**Keywords:** FLC, fusion *DNAJB1::PRKACA*, pediatric liver cancers

## Abstract

Arif and colleagues commented on our paper Fleifil et al. “DNAJB1-PKAc is expressed in Young Patients with Pediatric Liver Cancers and Enhances Carcinogenic Pathways” published in Cancers in 2024. In our paper, we examined expression of *DNAJB1::PRKACA* (DNAJB1-PKAc or J-PKAc) in the Bio Bank of HBL (Hepatoblastoma) and HCN-NOS (Hepatocellular Malignant Neoplasm, Not Otherwise Specified) tissue samples collected at CCHMC during the last five years. Our data demonstrated that *DNAJB1::PRKACA* was detected in approximately 70% of HBL/HCN-NOS patients, with varying expression levels. In the commentary, the authors reviewed their earlier data and found no evidence of the fusion DNAJB1-PKAc expression within their cohorts of HBL specimens. Based on these data, the authors stated that “…*DNAJB1::PRKACA* remains specific to fibrolamellar carcinoma among liver tumors and caution against its use as a diagnostic marker for hepatoblastoma without rigorous validation in external cohorts.” After reviewing the commentary, we are offering a response outlined below.

Key findings and interpretations from our Cancers paper.

In our study, we detected expression of *DNAJB1::PRKACA* in approximately 70% of children in our HBL/HCN-NOS and biliary atresia cohorts. Approximately 30% of examined patients did not show expression of *DNAJB1::PRKACA*. Within our patients, we identified two groups of fusion-positive pediatric HBL/HCN-NOS; one group had low levels of the fusion kinase compared to FLC, whereas the second group expressed *DNAJB1::PRKACA* at levels like FLC (Figure 1B, [Ref #1]) [1]. Thus, while about 70% of our non-FLC patients expressed *DNAJB1::PRKACA*, only some reached expression levels similar to FLC patients. We suggest that the detection of *DNAJB1::PRKACA* in non-FLC patients requires highly sensitive assays and may be missed if conditions are only optimized for strong fusion kinase levels, as seen in FLC. This may explain the differences between our findings and those of Arif et al. [2]. Arif’s commentary examined *DNAJB1::PRKACA* levels, comparing the generally high signals seen in FLC, to the low or undetectable levels in HBL patients. The detection sensitivity for low concentrations of *DNAJB1::PRKACA* described in the Commentary may be lower than that reported in our publication in Cancers [1]. Another reason for the discrepancies might be related to the patients’ ages at the time of *DNAJB1::PRKACA* analysis. As our research shows, accurately identifying *DNAJB1::PRKACA* in HBL patients from the ages of one to five years is crucial. Therefore, it is important to include more information about the HBL patients mentioned in the Commentary, especially their ages at the time *DNAJB1::PRKACA* was examined.

Important point of our paper is that comparisons of transcriptomic profiles in fusion-negative HBL and fusion-positive HBL with high expression of *DNAJB1::PRKACA* showed that “in young kids with fusion containing hepatoblastoma, J-PKAc (*DNAJB1::PRKACA*) worsens cancer by the activation of fibrosis, liver proliferation, and the massive repression of tumor suppressors”. Transcriptomic analysis also revealed that most FLC-specific genes display similar expression patterns in the DNAJB1:PRKACA positive HBL/HCN-NOS group (Figure 7, [Ref #1]). Our analysis of the *DNAJB1::PRKACA* targets in tissue culture systems showed that DNAJB1:PRKACA directly regulates these genes/pathways (Figure 8, [Ref #1]). These findings indicate that *DNAJB1::PRKACA* drives the observed alterations in gene expression, as identified by RNA-Seq analysis in the fusion-positive HBL/HCN-NOS samples.

Given that Arif et al. did not detect the fusion kinase in livers with non-FLC cohorts, the question is if the fusion *DNAJB1::PRKACA* kinase is only detectable in our non-FLC patients. According to the literature, the fusion kinase occurs in non-FLC patients. The authors of the commentary referred to the paper by Singhi et al. [Ref #3]) that “…the *DNAJB1::PRKACA* fusion was only identified in 26% of intraductal oncocytic papillary neoplasms of the pancreas and bile duct ...”. Singhi et al. reported six oncocytic papillary neoplasm cases, five with *DNAJB1::PRKACA* fusion [3]. The authors stated in the “Results” section that “these fusions were also detected in invasive PDACs, intrahepatic CCAs, pancreatic cyst fluid, and bile duct brushings. These gene rearrangements were absent from all 126 control pancreatobiliary lesions.” Notably, Weiel et al. identified a case of conventional hepatocellular carcinoma demonstrating positivity for *DNAJB1::PRKACA* via FISH assay with a specific probe. In this instance, 18% of the tumor cells exhibited fusion [4]. In addition, Rooper et al. identified *DNAJB1::PRKACA* in a sinonasal adenocarcinoma patient using an RNA-Seq approach [5].

Additional reports have documented the detection of *DNAJB1::PRKACA* in oncocytic pancreatic and biliary neoplasms. One of them is the manuscript by Vyas et al. [6]. Based on their data the authors conclude that “*DNAJB1::PRKACA* fusion is neither exclusive nor diagnostic for fibrolamellar hepatocellular carcinoma, and caution should be exercised in diagnosing liver tumors with DNAJB1-PRKACA fusions as fibrolamellar hepatocellular carcinoma”. Another report published by Itoh et al. in 2024 showed that *DNAJB1::PRKACA* is detected in seven (16%) patients with Oncocytic Pancreatobiliary Neoplasms (IOPNs) [7]. A separate study reported that *DNAJB1::PRKACA* was detected in 29% of IOPN cases (20 out of 68 analyzed) [8]. Maimaitiali et al. detected *DNAJB1::PRKACA* fusion in 27.8% of IOPN cases using RT-PCR-DNA sequencing [9]. Respectively, a recent review of IOPN by Qian et al. even highlighted *DNAJB1::PRKACA* fusion as one of the molecular hallmarks of IOPN [10]. Thus, including our Cancers paper, nine reports have identified DNAJB1-PRKACA in non-fibrolamellar cases [1,3,4,5,6,7,8,9,10]. Our findings in Cancer’s paper [1] support the Vyas et al. assertion that scientists need to exercise caution when diagnosing liver tumors with *DNAJB1::PRKACA* fusions as fibrolamellar hepatocellular carcinoma.

It is notable that some published studies have also found cases of FLC occurring without this mutation. Graham et al. reported in “Fibrolamellar Carcinoma in the Carney complex: PAKAR1aA loss instead of the classic *DNAJB1::PRKACA* fusion” [11] that PAKAR1aA loss can also be involved in FLC pathogenesis. The authors identified three cases of FLC lacking the *DNAJB1::PRKACA* mutation. This study questions if fusion DNAJB1-PRKACA is necessary for the development of FLC in patients. In this regard, a recent report indicated that the increase in Protein Kinase A activity leads to FLC characteristics regardless of the involvement of fusion component [12]. Moreover, the authors of this report mentioned that overexpression of PRKACA caused transcriptomic changes like those of *DNAJB1::PRKACA*. Thus, according to the published data, including findings from our study in Cancers, the involvement of fusion kinase in FLC as well as other cancers and neoplasms demonstrate considerable complexity. Detailed investigation of FLC cases without *DNAJB1::PRKACA* will clarify whether this fusion is a key driver of FLC.

The important question raised in the Arif et al. commentary is that there is “…need for rigorous validation before clinical interpretation of molecular findings in pediatric liver tumors” [2]. We concur, as stated in our Cancers paper that “The studies described in this manuscript were performed with available specimens from young patients with pediatric liver cancers and with biliary atresia. Future studies will be important to understand whether pediatric patients with severe liver cancer in other demographic areas have increased expression of J-PKAc…” (See Discussion in our Cancers paper [1]).

In summary: Several groups found *DNAJB1::PRKACA* in non-FLC cases [1,3,4,5,6,7,8,9,10], including our group for HBL and biliary atresia [1], whereas others such as Arif et al. did not [2]. It is possible that non-FLC patients who have increased fusion kinase are uncommon in certain demographic regions. An additional consideration is that the sensitivity of approaches to detect the fusion *DNAJB1::PRKACA* might differ among research groups, particularly when *DNAJB1::PRKACA* levels are low in certain non-FLC patients. These questions should be addressed in future studies using high-quality specimens and multiple sensitive methods to detect even small amounts of the fusion *DNAJB1::PRKACA* kinase. Given cases of FLC without the *DNAJB1::PRKACA* mutation, it is also important to understand if the mutant *DNAJB1::PRKACA* is required for FLC pathogenesis in patients or if changes in other pathways alone could cause FLC.

## Abbreviations

FLC, fibrolamellar hepatocellular carcinoma; HBL, hepatoblastoma; *DNAJB1::PRKACA*, DNAJB1-PKAc or J-PKAc; the fusion kinase.
